# Degradation of Indomethacin in Wastewater: Removal with Sodium Hypochlorite and Analysis of Degradation Byproducts

**DOI:** 10.3390/molecules30102180

**Published:** 2025-05-16

**Authors:** Antonio Medici, Giovanni Luongo, Lucio Previtera, Daniele Naviglio, Giovanni Di Fabio, Armando Zarrelli

**Affiliations:** 1 Department of Chemical Sciences, University of Naples Federico II, 80126 Naples, Italy; antonio.medici@unina.it (A.M.); giovanni.luongo@unina.it (G.L.); daniele.naviglio@unina.it (D.N.); difabio@unina.it (G.D.F.); 2 Associazione Italiana per la Promozione delle Ricerche su Ambiente e Salute umana, 82030 Dugenta, Italy; previter@unina.it

**Keywords:** indomethacin, sodium hypochlorite, oxidation processes, degradation byproducts, water treatment

## Abstract

Over the years, the frequent and continuous use of drugs has led to a high presence of emerging micropollutants in wastewater, increasing environmental and health concerns. Among these chemicals, Indomethacin (IND), a widely used anti-inflammatory drug, has been detected up to 150 ng/L in water bodies. Its presence in aquatic environments causes increasing concerns due to its high persistence, limited biodegradability, and resistance to conventional treatment processes. This study examined the degradation of IND via oxidation with sodium hypochlorite (NaClO) and the characterization of the degradation byproducts (DPs) generated by this process. Based on NMR spectroscopy studies and mass spectrometry analysis, thirteen DPs were identified, seven of which were previously unpublished (DP1: 2-(3-Chloro-1-(4-chlorobenzoyl)-2-hydroxy-5-methoxy-2-methylindolin-3-yl)acetic acid, DP3: 2-(3,4-Dichloro-1-(4-chlorobenzoyl)-2-hydroxy-5-methoxy-2-methylindolin-3-yl)acetic acid, DP5: (3-Chloro-5-methoxy-2-methyl-1H-indol-1-yl)(4-chlorophenyl)methanone, DP6: (4-Chlorophenyl)(5-methoxy-3-(methoxymethyl)-2-methyl-1H-indol-1-yl)methanone, DP7: 2-(2-(4-Chlorobenzamido)-5-methoxyphenyl)-2- oxoethyl acetate, DP8: 2-(5-Methoxy-2-methyl-1H-indol-3-yl)acetic acid, DP9: 4-Chloro-*N*-(4-methoxyphenyl)benzamide), and a degradation mechanism was proposed. These results show how the degradation of Indomethacin leads to the generation of new byproducts that may persist in the environment, obtaining DP1 in far larger quantities than the other byproducts. Given Indomethacin’s degradation rate of over 90% but not its complete mineralization, it is fundamental to study not only IND but also the byproducts generated to assess their potential environmental impact.

## 1. Introduction

In recent years, a plethora of chemical compounds have been identified in supply waters and treated effluents, derived largely from human and animal antibiotics, prescriptions, and generic drugs [1], sex and steroid hormones [2], and even industrial and civil wastewater products [3]. A common characteristic of these pollutants is that they are present in water at very low concentrations in the order of ng/L, or few µg/L at most, and are therefore also called micropollutants. Most of these compounds have been classified as emerging pollutants, since many are not yet regulated [4] and have chemical and physical properties that can induce adverse effects on many organisms, particularly aquatic ones. In fact, the environmental implications are well documented [5], albeit without excluding adverse effects on humans, such as allergies or the selection of antibiotic-resistant bacterial strains [6]. Reducing the presence of these substances in the environment by limiting their use is difficult, especially for pharmaceuticals. Thus, a fundamental role in the control of environmental pollution by micropollutants is assumed by sewage treatment plants. Wastewater treatment plants, however, are not designed to remove such complex substances so different from one other and at such low concentrations. As a result, many of them are not completely degraded and therefore remain in the treated wastewater, spilling out and accumulating in the environment. Thus, there is the need to improve existing treatment units in plants or implement them with new technologies [7,8,9], particularly by studying and evaluating the most efficient advanced oxidation processes [10,11,12] that allow greater mineralization and fewer byproducts compared to hypochlorite treatment, one of the most widely used in the world now. One of the main challenges in wastewater treatment is that degradation byproducts formed during treatment are not necessarily less toxic than the parent compounds [13,14,15].

**Figure 1:**
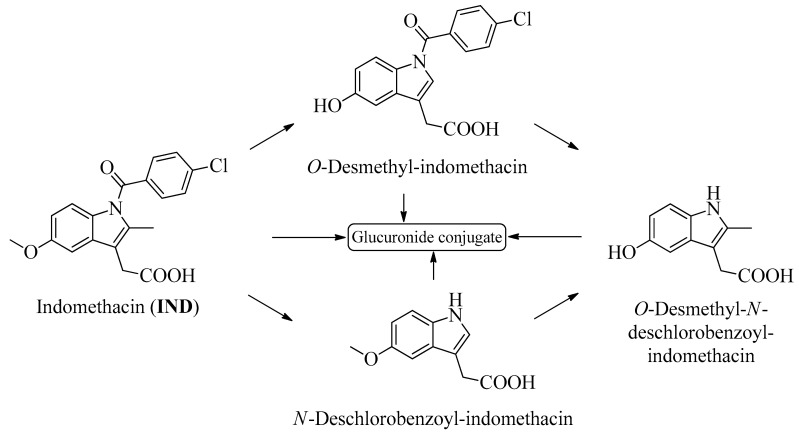
Metabolism of Indomethacin in humans [26].

Worsening the situation is the COVID-19 epidemic, which has dramatically increased pharmaceutical consumption on a global scale. Thus, increasing concentrations of analgesic and anti-inflammatory drugs (AAIDs) in wastewater [16,17,18,19], surface water [20], and sometimes even drinking water [21] are increasingly becoming a problem for human and aquatic life [22,23,24]. Since the long-term effect of these substances in the environment is not yet well known, it is important to reduce their presence by mitigating the pollution caused by their occurrence in WWTP waters. Therefore, simple and effective treatment options are needed to remove AAIDs, such as Indomethacin, from wastewater [25]. Indomethacin (IND) is a non-steroidal, anti-inflammatory drug (NSAID) with analgesic and antipyretic properties. It is an indoleacetic acid derivative with the chemical name 2-(1-(4-Chlorobenzoyl)-5-methoxy-2-methyl-1H-indol-3-yl) acetic acid (Figure 1). The pharmacological effect of Indomethacin is not completely understood. However, it is believed to be mediated by the potent and non-selective inhibition of the enzyme cyclooxygenase (COX) [26], which is the main enzyme responsible for catalyzing the rate-limiting step in the biosynthesis of prostaglandins [27]. It was discovered in 1963, and it was first approved for use by the US Food and Drug Administration in 1965. Nowadays, it is included on the World Health Organization’s list of essential medicines. In 2022, it was the 256th most prescribed drug in the United States, with over 1 million prescriptions [28]. In the same year, it is estimated that just over 60 tons were produced in China, nearly double the previous year’s output [29].

This drug has been widely used in the symptomatic treatment of migraine and headaches, as well as in the treatment of rheumatoid arthritis, ankylosing spondylitis, osteoarthritis, acute shoulder pain, and acute gouty arthritis [30]. In the human organism, IND is largely converted into inactive metabolites by metabolic reactions including demethylation, deacylation, and glucuronidation (approximately 10% of a dose) [31]. The main metabolites include *O*-desmethyl-indomethacin, *O*-deschlorobenzoyl–indomethacin, *O*-Desmethyl-*N*-deschlorobenzoyl-indomethacin, and their glucuronide conjugates, which are then excreted via the urine and feces, together with 10–20% of the drug in an unmodified form [32].

Indomethacin is not completely removed during treatment in wastewater treatment plants (WWTPs), and the effluents are released into aquatic environments. Indeed, in Canada, concentrations of Indomethacin of less than 10 ng/L were detected in the Little River, which receives effluent from a WWTP [33]. Concentrations of just under 70 ng/L were measured in Lake Tegel in Germany [34]; concentrations of up to 151 ng/L were measured in the tributaries of a nearby WWTP and up to 91 ng/L in the effluent. Also in Germany, average concentrations of 0.27 μg/L were measured in the effluents of a different WWTP, up to a maximum of 0.60 μg/L [35].

Various studies have demonstrated the degradation of IND by various advanced oxidation processes, by photocatalytic processes [36,37,38], by a based ferrous–peroxydisulfate oxidative system [39], and using UV-vis/peroxydisulfate [40] that resulted in the complete degradation of the drug in less than 30 min. But to the best of our knowledge, there are no studies in the literature showing the degradation of IND by hypochlorite. To date, there are no wastewater treatment processes for the removal of pharmaceuticals widely used, but only disinfection processes, most often using hypochlorite. While effective in eliminating microbial contaminants, hypochlorite may react with pharmaceutical pollutants such as IND, which are often present in effluents, to form disinfection byproducts. These byproducts can contain highly reactive and potentially toxic functional groups, raising concerns about their environmental fate and impact. Therefore, in this study, we focused on the degradation of IND by hypochlorite and the identification of its possible byproducts. The efficiency of its removal was determined to be around 95% with the formation of 13 disinfection byproducts (DPs) in percentages ranging from 0.38 to 13.26% by weight of the parent product.

Finally, a possible reaction mechanism was formulated to explain the obtaining of the isolated products and other potential byproducts that have not yet been identified, later comparing them with those identified using different degradation techniques.

## 2. Results and Discussion

### 2.1. Degradation Experiments

**Figure 2:**
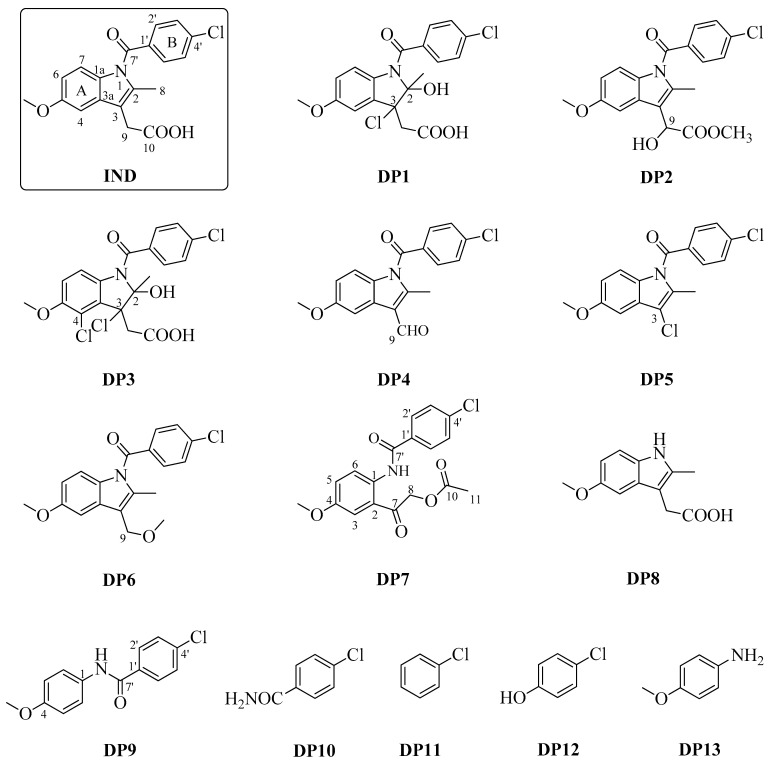
Chemical structures of Indomethacin and its degradation byproducts.

Indomethacin was oxidized in an aqueous suspension at a concentration of approximately 10^−5^ M for 10 min (IND:NaClO molar ratio of 1:1), under stirring, and at room temperature (analytical conditions). The duration of the experiment is related to the complete degradation of the product or its maximum conversion into the corresponding DPs (Figure 2). Subsequently, suspensions of concentrations at least a hundred times greater, with an IND:NaClO molar ratio of 1:20, were considered, again under stirring and at room temperature (preparative conditions). Thus, DPs obtained under both analytical and preparative conditions were purified by column chromatography, TLC and HPLC, followed by point characterization by NMR techniques (^1^H, ^13^C, COSY, HSQC, HMBC, NOESY) and MALDI-TOF experiments.

DP1–DP13 were isolated in quantities of 13.26, 1.70, 2.08, 0.38, 0.38, 1.14, 0.38, 1.14, 2.46, 2.84, 3.79, 2.27, and 1.33%, respectively.

Of the 13 compounds identified, DP1, DP3, and DP5–DP9 were new; DP2 had been prepared by modifying Indomethacin in search of new, more potent anti-inflammatory compounds [41]; DP4 was identified by comparing the NMR data with those of the same compound obtained by photodegrading Indomethacin in aqueous solution involving UV light or sunlight [42,43]; DP10–DP13 were identified by comparing their data with those of the corresponding commercially available compounds. The total byproduct recovery rate was 33%, while the degradation of the starting molecule was more than 90%. The difference between these two values is substantially related to different factors. Since IND has a very high reactivity towards hypochlorite, with a degradation of more than 90% while involving byproducts, it is probable that part of the missing mass is due to byproducts that were not completely extracted from the reaction solution, or to extremely polar and water-soluble byproducts that could not be isolated by the techniques used in this study. It is also possible that compounds of sufficiently low molecular weight to escape in the gas phase from the reaction mix are formed.

### 2.2. Mechanistic Interpretation

**Scheme 1:**
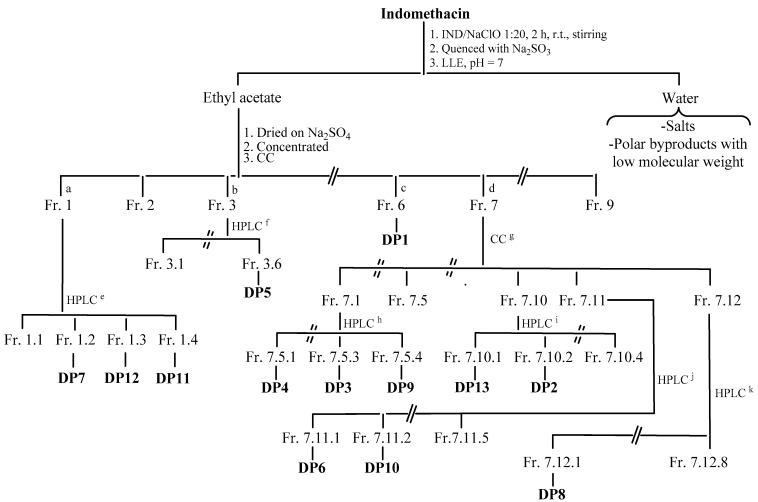
Isolation of the byproducts DP1–DP13. ^a^ Petroleum ether:acetone:acetic acid (95:5:0.5, *v*/*v*/*v*); ^b^ Petroleum ether:acetone:acetic acid (90:10:0.5, *v*/*v*/*v*); ^c^ Petroleum ether:acetone:acetic acid (80:20:0.5, *v*/*v*/*v*); ^d^ Petroleum ether:acetone:acetic acid (70:30:0.5, *v*/*v*/*v*); ^e^ CH_3_COONH_4_ (A, pH 4.0; 20 mM) and acetonitrile (B, 95:5 to 5:95); ^f^ HCOOH (C, 0.1% in H_2_O) and methanol (D, 95:5 to 5:95); ^g^ Petroleum ether:ethyl acetate:acetic acid (95:5:0.5 to 60:40:0.5, *v*/*v*/*v*); ^h^ HCOONH_4_ (E, pH 3.0; 15 mM) and B (95:5 to 5:95); ^i^ C and B (90:10 to 0:100); ^j^ A and D (95:5 to 5:95); ^k^ E and D (90:10 to 0:100).

**Figure 3:**
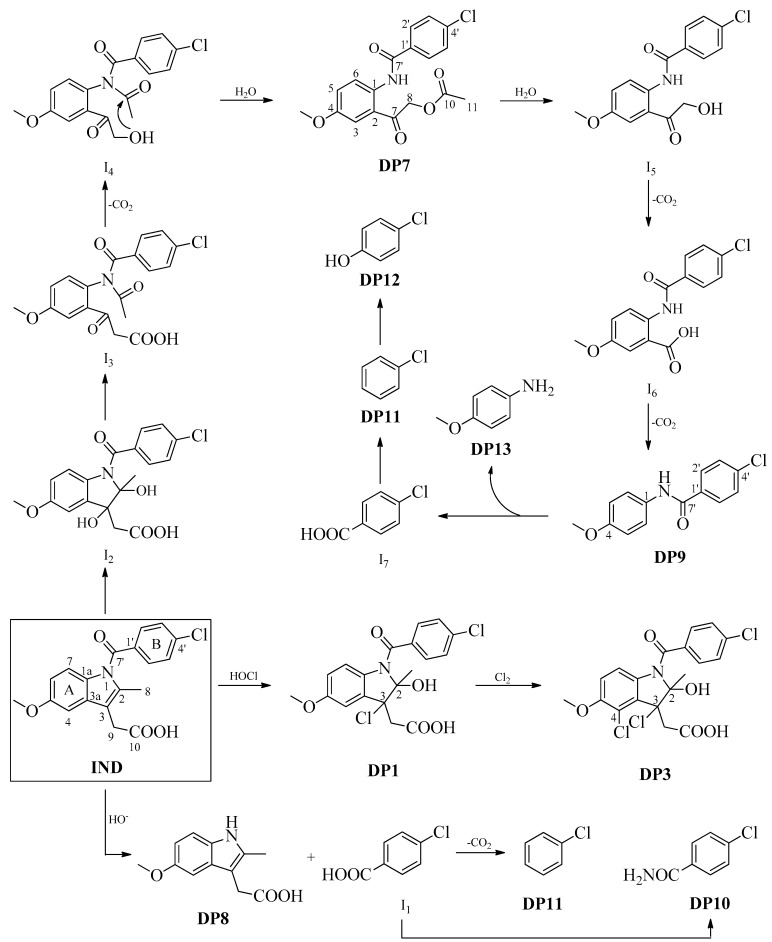
Plausible mechanism for the formation of DP1, DP3, DP7–DP13.

**Figure 4:**
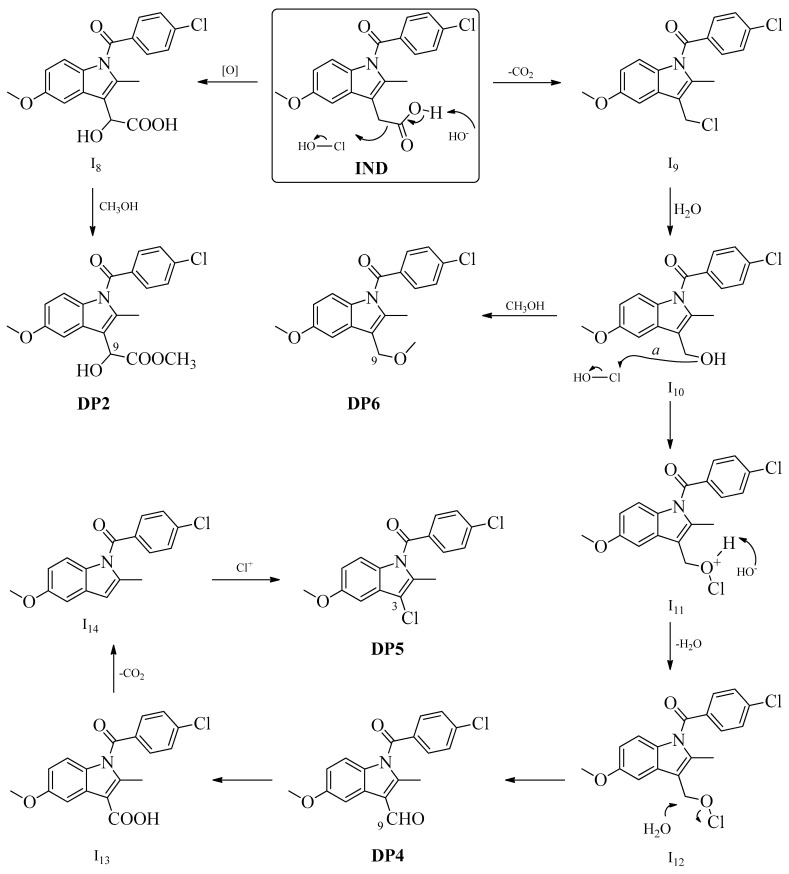
Plausible mechanism for the formation of DP2, and DP4–DP6.

Oxidation reactions were conducted at pH close to neutrality, as in the wastewater treatment plant. The formation of DPs was followed over time by HPLC monitoring. Of the 13 degradation products obtained from the oxidation of Indomethacin, 12 were formed under both analytical and preparative conditions (Figure 1). Scheme 1 indicates from which fractions the different DPs were obtained, while Figure 3 and Figure 4 show a plausible mechanism for their formation.

Indomethacin and the DP1–DP6 products show two phenyl aromatic rings: the 1,2,4-trisubstituted A-ring and the 1,4-disubstituted B-ring. Both rings remained unchanged apart from the chlorination of the C4 position of ring A in DP3.

The addition of HOCl on the C2-C3 bond would lead to DP1, from which DP3 would be obtained by chlorination of the C4 position of the aromatic ring A (Figure 3). Hydrolysis of the C7′-N amide bond would explain the formation of DP8 and the intermediate I_1_. The latter would give DP11 by decarboxylation and DP10 by amidation.

The oxidation of the C2-C3 bond would first lead to the formation of the intermediate I_2_ and then the intermediate I_3_, resulting in the complete cleavage of this bond. The oxidative decarboxylation of intermediate I_3_ would lead to the formation of intermediate I_4_, in which an intramolecular attack could occur with the formation of the product DP7. It is easy to imagine that DP7 would then undergo deacetylation, i.e., the hydrolysis reaction of the ester bond, and lead to the formation of I_5_, by which a double decarboxylation reaction would yield DP9. Hydrolysis of the amide bond of the latter would yield the product DP13 and 4-chlorobenzoic acid (I_7_), from which DP11 could be obtained by decarboxylation, and oxidation of the aromatic ring of the latter would yield DP12, (Figure 3).

Indomethacin could undergo oxidation at carbon C9, in alpha to the carboxyl group, and give the intermediate I_8_ (Figure 4), by which methylation of the carboxyl function itself would yield DP2. Indomethacin could also undergo loss of carboxyl function and yield the chlorine derivative I_9_, which could then be oxidized to the corresponding alcohol I_10_. The latter, by methylation of the alcohol function, would yield DP6 and, by oxidation, DP4 through possible intermediates I_11_ and I_12_. Subsequent oxidation of DP4 would yield the intermediate I_13_, which would then, by decarboxylation and subsequent addition of chlorine, yield DP5.

### 2.3. Kinetic Study of the Effect of Hypochlorite Concentration

To understand the degradation mechanism of Indomethacin in the presence of sodium hypochlorite, different experiments at different hypochlorite concentrations (Table 1) were conducted to evaluate the efficiency of the process. The results obtained are reported below, with particular emphasis on the degradation rate and the influence of the sodium hypochlorite (NaClO) concentration on the process. For each hypochlorite concentration test, experiments were conducted in triplicate. Kinetic experiments were performed using three different NaClO concentrations to assess the kinetic behavior of the reaction between IND and NaClO. In all experiments performed, degradation rates very close to 100% were obtained, which demonstrates the high reactivity of Indomethacin towards hypochlorite, and thus the need for environmental control of its byproducts. Based on these data, the most efficient concentration of the oxidant for the degradation of IND was calculated. To assess the kinetics of drug degradation in the presence of NaClO, experiments were conducted using three different NaClO concentrations. Fixed initial drug concentration (3.00 mM) and final concentration after a 2 h treatment were measured and reported in Table 1.

**Figure 5:**
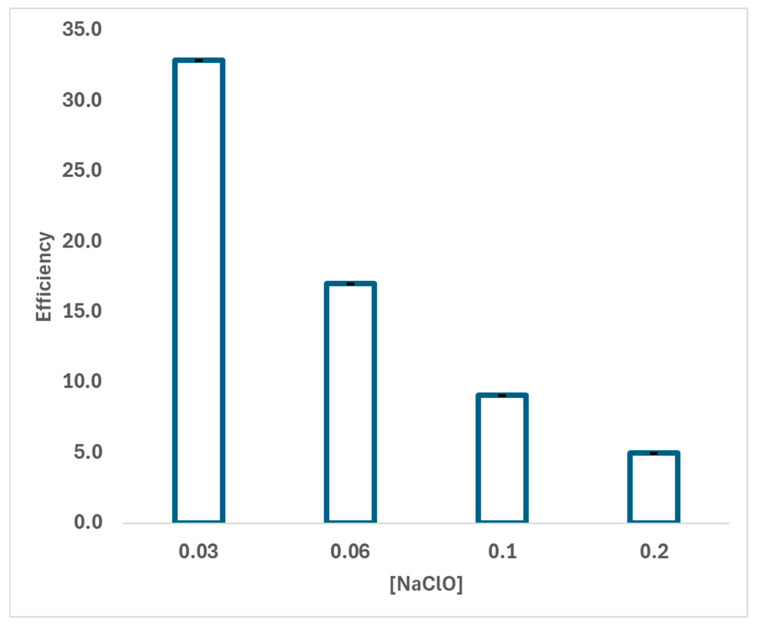
Plotting of efficiency values as a function of different NaClO concentrations.

The degradation efficiency parameter was defined as

$$Efficency=\frac{\left(\frac{{\left[C\right]}_{0}-{\left[C\right]}_{f}}{{\left[C\right]}_{0}}\right)}{\left[NaClO\right]}$$

where [C]_0_ and [C]_f_ represent the initial and final concentration of indomethacin, respectively, and [NaClO] indicates the concentration of hypochlorite used for degradation experiments. The standard deviation of the efficiency was calculated across the triplicates for each hypochlorite concentration, providing a measure of the experimental variability (Figure 5). These standard deviations were incorporated into the reported efficiency values to indicate the statistical uncertainty associated with each measurement.

The results obtained show that although all NaClO concentrations tested resulted in the almost complete degradation of Indomethacin, the efficiency of the process varies depending on the concentration of the oxidant. In particular, the highest efficiency value was recorded at a concentration of 0.03 M NaClO, indicating that this is the most advantageous condition in terms of consumption of the oxidant relative to the amount of drug degraded. Analyzing the data, it is observed that as the NaClO concentration increases, the degradation rate increases slightly while efficiency decreases progressively. This behavior suggests that higher concentrations of NaClO do not provide a significant benefit in terms of abatement of the target substance, but leads to an increase in reagent consumption, making the process environmentally and economically sustainable.

### 2.4. Environmental Persistence and Toxicological Significance of Degradation Products

Quite a few of the DPs identified in this study (notably DP1, DP3, and DP5–DP9) were formerly unreported and essentially distinct from the parent compound, Indomethacin. These DPs, formed through oxidation, hydrolysis, and chlorination reactions, may exhibit variable environmental fate and biological activity compared to the parent drug. In general, however, several literature studies show the increased toxicity of byproducts and effluents generated by hypochlorite treatment [44,45,46].

In particular, the formation of chlorinated byproducts such as DP1, DP3, and DP5 may lead to high concerns over their persistence in the environment and higher toxicity, especially given their well-documented environmental persistence due to their low biodegradability and limited photodegradation [13,43,47]. Furthermore, these byproducts may also induce endocrine disruption and cytotoxicity effects as suggested in similar research on chlorinated pharmaceuticals and DPs [48,49]. DP4 displays an aldehyde functional group, which is well known to exhibit high chemical reactivity. It can form covalent adducts such as Schiff bases through reactions with nucleophilic moieties present in biomolecules, including amino groups in proteins and nucleic acids, leading to their well-documented cytotoxic and genotoxic effects [50].

## 3. Materials and Methods

### 3.1. Drug and Reagents

Indomethacin (99%) was purchased from Merck (Darmstadt, Germany). Solvents were purchased from Merck (Darmstadt, Germany), of an HPLC grade, and used as received. All other chemicals were of an analytical grade and were supplied by Merck, Darmstadt, Germany.

### 3.2. Sodium Hypochlorite Reaction

#### 3.2.1. Apparatus and Equipment

Kieselgel 60 (230–400 mesh, Merck, Darmstadt, Germany) was used for column chromatography (CC). HPLC analysis utilized a Shimadzu LC-8A system equipped with a Shimadzu SPD-10A VP UV-vis detector (Shimadzu, Milan, Italy). NMR spectra (^1^H and ^13^C) were recorded at 400 MHz and 25 °C (Bruker DRX, Bruker Avance, Billerica, MA, USA), and the results were referenced to residual solvent signals (CDCl_3_, δ_H_ 7.27 and δ_C_ 77.0; CD_3_OD, δ_H_ 3.30 and δ_C_ 49.0). Proton-detected heteronuclear correlations were measured using gradient heteronuclear single-quantum coherence (HSQC), optimized for ^1^J_HC_ = 155 Hz, and gradient heteronuclear multiple-bond correlation (HMBC), optimized for ^n^J_HC_ = 8 Hz. MALDI-TOF mass spectrometric analyses were conducted on a Voyager-De Pro MALDI mass-spectrometer (PerSeptive Biosystems, Framingham, MA, USA). UV-vis analysis was performed on a Lambda 12 UV-vis spectrophotometer (Perkin Elmer, Waltham, MA, USA). The lyophilization of samples was performed using a LyovaporTM-200 (Buchi, Cornaredo (MI), Italy), with a compressor featuring a cooling capacity of 1.97 kW for 50 Hz and a minimum condenser temperature of −55 °C.

#### 3.2.2. Chlorination Reaction

A 10^−5^ M solution of IND was exposed to a 10% hypochlorite solution for 10 min at room temperature, maintaining a molar ratio of IND to hypochlorite of 1:1. The presence of IND was determined spectrophotometrically with absorbance peaks measured at 240 nm [51]. The recorded absorbance values were subsequently converted into concentration values using a calibration curve generated from standard IND solutions of known concentrations. Although DP1–DP13 (Figure 1) were formed under these conditions, their abundance was too low for isolation. However, their retention times were assessed by comparison with the byproducts generated from IND degradation in the preparative experiments described later. DPs common to both the analytical and preparative experiments were then isolated from the ethyl acetate extract of the aqueous solution.

Preparative experiments were carried out using an IND solution with a concentration slightly above 10^−3^ M, specifically 1.5 × 10^−3^ M, suspended in water. A 10% hypochlorite solution was added while stirring magnetically at room temperature. The reaction was monitored by taking samples every 15 min, quenching with an excess of sodium sulphite, and subsequently drying under vacuum. The resulting residue was dissolved in a saturated sodium bicarbonate solution, extracted with ethyl acetate, and analyzed by HPLC. The reaction was terminated after 120 min to ensure maximal degradation of IND and formation of its DPs by quenching with sodium sulphite excess and concentrating by lyophilization. The residue was dissolved in water and pH-adjusted to 7.0 before extraction with ethyl acetate. The extraction yielded an organic fraction of 548 mg, which was chromatographed to obtain DPs.

#### 3.2.3. Product Isolation Procedure

The organic fraction obtained from the preparative experiment (548 mg) was subjected to chromatographic separation with a gradient of petroleum ether:acetone:acetic acid (ranging from 100:0:0.5 to 60:40:0.5, *v/v/v*) on silica gel to yield 9 fractions. Fraction 1 (Fr. 1), obtained from elution with petroleum ether:acetone:acetic acid 95:5:0.5, *v/v/v*, was further purified via HPLC under a gradient condition, which involved CH_3_COONH_4_ (pH 4.0; 20 mM) and acetonitrile (from 95:5 to 5:95). This was carried out on a Phenomenex Kromasil 10 μm 100 Å C18 (250 × 10.00 mm) HPLC column at a solvent flow rate of 5 mL/min, obtaining four subfractions, of which the last three were DP7 (2 mg), DP12 (12 mg), and DP11 (20 mg), respectively. Fraction 3 (Fr. 3), obtained from elution with petroleum ether:acetone:acetic acid 90:10:0.5, *v/v/v*, underwent further purification using HPLC under gradient condition *g* of HCOOH (0.1% in H_2_O) and methanol (from 95:5 to 5:95). This purification step was conducted on a Phenomenex Prodigy 10 μm ODS (250 × 10.00 mm) HPLC column at a solvent flow rate of 5 mL/min, obtaining six subfractions, of which the last one contained DP5 (2 mg). Fraction 6 (Fr. 6), obtained from elution with petroleum ether:acetone:acetic acid 80:20:0.5, *v/v/v*, contained DP1 (70 mg). Fraction 7 (Fr. 7), obtained from elution with petroleum ether:acetone:acetic acid 70:30:0.5, *v/v/v*, underwent subsequent purification via column chromatography (CC) and eluted with petroleum ether:ethyl acetate:acetic acid (from 95:5:0.5 to 60:40:0.5, *v/v/v*), resulting in 12 subfractions. Subsequently, subfraction 7.5 was further purified via HPLC under gradient condition *h*, entailing HCOONH_4_ (pH 3.0; 15 mM) and acetonitrile (from 95:5 to 5:95). This was performed on Phenomenex Synergi 10 μm 110 Å C18 (250 × 10.00 mm) column at a solvent flow rate of 5 mL/min, yielding DP3 (11 mg), DP4 (2 mg), and DP9 (13 mg). Subfraction 7.10 was purified via HPLC using gradient condition *i*, involving HCOOH (0.1% in H_2_O) and acetonitrile (from 90:10 to 0:100). This process utilized a Phenomenex Luna 5 μm C18 100 Å (250 × 10.00 mm) HPLC column at a solvent flow rate of 5 mL/min, obtaining four subfractions, of which the first two were DP13 (7 mg) and DP2 (9 mg). Subfraction 7.11 was purified via HPLC under gradient condition *j*, entailing CH_3_COONH_4_ (pH 4.0; 10 mM) and methanol (from 95:5 to 5:95). This was conducted on the same column used for Fr. 3, obtaining five subfractions, of which the first two were DP6 (6 mg) and DP10 (15 mg). Finally, subfraction 7.12 was purified via HPLC under the gradient condition *k*, HCOONH_4_ (pH 3.0; 15 mM), and methanol (from 90:10 to 0:100). We used the same column used for Fr. 7.5, obtaining eight subfractions, of which the first one was DP8 (6 mg).

### 3.3. Spectral Data

IND: 2-(1-(4-Chlorobenzoyl)-5-methoxy-2-methyl-1H-indol-3-yl)acetic acid. White powder. NMR data see Table S1. MS-TOF (positive ions): *m*/*z* calculated for C_19_H_16_ClNO_4_ 357.08 [M]^+^; found 358.79 [M + H]^+^ (84%), 360.79 [M + H + 2]^+^ (25%)

DP1: 2-(3-Chloro-1-(4-chlorobenzoyl)-2-hydroxy-5-methoxy-2-methylindolin-3-yl) acetic acid. Gray powder. NMR data see Table S2. MS-TOF (positive ions): *m*/*z* calculated for C_19_H_17_Cl_2_NO_5_ 409.05 [M]^+^; found 410.25 [M + H]^+^ (30%), 412.25 [M + 2 + H]^+^ (17%), 414.25 [M + 4 + H]^+^ (2%).

DP2: Methyl 2-(1-(4-chlorobenzoyl)-5-methoxy-2-methyl-1H-indol-3-yl)-2-hydroxy acetate. Gray powder. NMR data see Table S3. MS-TOF (positive ions): *m*/*z* calculated for C_20_H_18_ClNO_5_ 387.09 [M]^+^; found 388.81 [M + H]^+^ (35%), 390.81 [M + 2 + H]^+^ (10%).

DP3: 2-(3,4-Dichloro-1-(4-chlorobenzoyl)-2-hydroxy-5-methoxy-2-methylindolin-3- yl)acetic acid. Gray powder. NMR data see Table S4. MS-TOF (positive ions): *m*/*z* calculated for C_19_H_16_Cl_3_NO_5_ 443.01 [M]^+^; found 444.69 [M + H]^+^ (70%), 445.70 [M + 1 + H]^+^ (14%), 446.70 [M + 2 + H]^+^ (69%), 447.70 [M + 3 + H]^+^ (13%), 448.71 [M + 4 + H]^+^ (22%).

DP4: 2-(1-(4-Chlorobenzoyl)-5-methoxy-2-methyl-1H-indol-3-yl)acetaldehyde. Gray powder. NMR data see Table S5. MS-TOF (positive ions): *m*/*z* calculated for C_19_H_16_ ClNO_3_ 341.08 [M]^+^; found 342.79 [M + H]^+^ (44%), 343.79 [M + 2+ H]^+^ (13%).

DP5: (3-Chloro-5-methoxy-2-methyl-1H-indol-1-yl)(4-chlorophenyl)methanone. NMR data see Table S6. MS-TOF (positive ions): *m*/*z* calculated for C_17_H_13_Cl_2_NO_2_ 333.03 [M]^+^; found 334.20 [M + H]^+^ (40%), 336.20 [M + 2 + H]^+^ (22%), 338.21 [M + 4 + H]^+^ (5%).

DP6: (4-Chlorophenyl)(5-methoxy-3-(methoxymethyl)-2-methyl-1H-indol-1-yl)me thanone. Gray powder. NMR data see Table S7. MS-TOF (positive ions): *m*/*z* calculated for C_19_H_18_ClNO_3_ 343.10 [M]^+^; found 344.80 [M + H]^+^ (51%), 346.81 [M + 2 + H]^+^ (13%).

DP7: 2-(2-(4-Chlorobenzamido)-5-methoxyphenyl)-2-oxoethyl acetate. Gray powder. NMR data see Table S8. MS-TOF (positive ions): *m*/*z* calculated for C_18_H_16_ClNO_5_ 361.07 [M]^+^; found 362.78 [M + H]^+^ (75%), 364.78 [M + 2 + H]^+^ (23%).

DP8: 2-(5-Methoxy-2-methyl-1H-indol-3-yl)acetic acid. Gray powder. NMR data see Table S9. MS-TOF (positive ions): *m*/*z* calculated for C_12_H_13_NO_3_ 219.09 [M]^+^; found 220.24 [M + H]^+^ (71%).

DP9: 4-Chloro-*N*-(4-methoxyphenyl)benzamide. Gray powder. NMR data see Table S10. MS-TOF (positive ions): *m*/*z* calculated for C_14_H_12_ClNO_2_ 261.06 [M]^+^; found 262.70 [M + H]^+^ (60%), 264.70 [M + 2 + H]^+^ (19%).

DP10: Chlorobenzamide. Oily liquid. The compound was identified by comparison with an authentic, commercially available sample.

DP11: Chlorobenzene. Oily liquid. The compound was identified by comparison with an authentic, commercially available sample.

DP12: 4-Chlorophenol. Oily liquid. The compound was identified by comparison with an authentic, commercially available sample.

DP13: 4-Methoxyaniline. Oily liquid. The compound was identified by comparison with an authentic, commercially available sample.

## 4. Conclusions

In conclusion, our study sheds light on the concerning presence and potential risks associated with Indomethacin contamination in aquatic environments. The use of this drug in large quantities has led to an increase in its environmental concentration, but the effects of the multitude of byproducts that can be formed via the classic wastewater treatment such as hypochlorite treatment are currently unidentified, as demonstrated in this work. There were 13 isolated byproducts, and their structure was determined by NMR and mass spectrometry studies. The kinetic study showed that the most efficient condition for degradation was obtained with 0.03 M NaClO, demonstrating that an excess of hypochlorite does not significantly improve degradation but reduces the efficiency of the process. These degradation byproducts, obtained in percentages between 0.38 and 13.2% and for a total yield just over 33%, provide a better understanding of the reaction mechanisms that lead to the formation of these byproducts and can give valuable insights into byproducts that could be formed from similar molecules or from the same family. These insights contribute to our understanding of the possible fate of drugs in the environment but especially of their byproducts, whose chemical structures and environmental effects are unknown, hence the importance of limiting their formation and reducing their environmental presence.

## Figures and Tables

**Table 1:** Effects of NaClO concentration on compound degradation.

Initial Concentration (mM)	Final Concentration (mM)	NaClO Concentration (M)	Efficiency (M^−1^)
3.00	1.50 ± 0.22 ∙ 10^−3^	0.06	16.6583 ± 0.0013
3.00	1.61 ± 0.24 ∙ 10^−3^	0.03	33.3155 ± 0.0026
3.00	1.91 ± 0.29 ∙ 10^−3^	0.1	9.9936 ± 0.0010
3.00	1.11 ± 0.17 ∙ 10^−3^	0.2	4.9981 ± 0.0003

## Data Availability

Data are contained within the article and Supplementary Materials.

## Supplementary Materials

The following supporting information can be downloaded at: https://www.mdpi.com/article/10.3390/molecules30102180/s1, Table S1: Data obtained from mono- and two-dimensional NMR analysis of Indomethacin in deuterated chloroform (CDCl_3_); Table S2: Data obtained from mono- and two-dimensional NMR analysis of DBP1 in deuterated chloroform (CDCl_3_); Table S3: Data obtained from mono- and two-dimensional NMR analysis of DBP2 in deuterated chloroform (CDCl_3_); Table S4: Data obtained from mono- and two-dimensional NMR analysis of DBP3 in deuterated chloroform (CDCl_3_); Table S5: Data obtained from mono- and two-dimensional NMR analysis of DBP4 in deuterated chloroform (CDCl_3_); Table S6: Data obtained from mono- and two-dimensional NMR analysis of DBP5 in deuterated chloroform (CDCl_3_); Table S7: Data obtained from mono- and two-dimensional NMR analysis of DBP6 in deuterated chloroform (CDCl_3_); Table S8: Data obtained from mono- and two-dimensional NMR analysis of DBP7 in deuterated chloroform (CDCl_3_); Table S9: Data obtained from mono- and two-dimensional NMR analysis of DBP8 in deuterated chloroform (CDCl_3_); Table S10: Data obtained from mono- and two-dimensional NMR analysis of DBP9 in deuterated chloroform (CDCl_3_).
