# Supplementary material for: Degradation of Indomethacin in Wastewater: Removal with Sodium Hypochlorite and Analysis of Degradation Byproducts
Source: Molecules. 2025 May 16;30(10):2180. doi: 10.3390/molecules30102180 (PMC12114100; doi:10.3390/molecules30102180)
Supplement: Supplementary file 1 [file molecules-30-02180-s001.zip › molecules-3567109-supplementary.pdf]

Table S1. Data obtained from mono- and two-dimensional NMR analysis of Indomethacin in deuterated chloroform ($CDCl_3$).

Position	Residue	$^{13}C^{a}$	$^{1}H^{a}$, multiplicity (J in Hz)	^{1}H-^{1}H COSY	^{1}H-^{13}C HMBC
2	C	136.19			
3	C	111.88			
4	CH	101.24	6.94, d (2.4)	6.67	130.75, 130.46, 111.88, 111.65
5	C	156.03			
6	CH	111.65	6.67, dd (9.0; 2.6)	6.94, 6.85	130.75, 101.24
7	CH	114.97	6.85, d (8.8)	6.67	156.03, 130.46
8	CH_3	13.27	2.38, s		136.19, 111.88
9	CH_2	29.99	3.69, s		176.54, 136.19, 130.46, 111.88
10	C	176.54			
1a	C	130.75			
3a	C	130.46			
5-OCH_3	CH_3	55.69	3.82, s		156.03
1′	C	133.77			
2′/6′	CH	131.16	7.66, d (8.0)	7.46	168.28, 139.31, 131.16
3′/5′	CH	129.11	7.46, d (8.4)	7.66	139.31, 133.77, 129.11
4′	C	139.31			
7′	C	168.28			

[a] Chemical shifts in ppm.

Table S2. Data obtained from mono- and two-dimensional NMR analysis of **DBP1** in deuterated chloroform ($CDCl_3$).

Position	Residue	$^{13}C^{a}$	$^{1}H^{a}$, multiplicity (J in Hz)	^{1}H-^{1}H COSY	^{1}H-^{13}C HMBC
2	C	104.37			
3	C	71.58			
4	CH	109.11	6.97, d (2.5)	6.76	134.39, 130.71, 117.13, 71.58
5	C	157.20			
6	CH	117.13	6.76, dd (9.1; 2.5)	6.97, 6.70	134.39, 109.11
7	CH	117.79	6.70, d (9.1)	6.76	157.20, 130.71
8	CH_3	21.23	1.98, s		
9	CH_2	42.79	3.36, d (17.7)	3.50	168.80, 130.71, 104.37, 71.58
			3.50, d (17.7)	3.36	168.80, 130.71, 104.37, 71.58
10	C	168.80			
1a	C	134.39			
3a	C	130.71			
5-OCH_3	CH_3	55.68	3.79, s		157.20
1′	C	133.65			
2′/6′	CH	130.07	7.65, d (8.3)	7.44	167.69, 138.35, 130.07
3′/5′	CH	129.02	7.44, d (8.3)	7.65	138.35, 133.65, 129.02
4′	C	138.35			
7′	C	167.69			

[a] Chemical shifts in ppm.

Table S3. Data obtained from mono- and two-dimensional NMR analysis of **DBP2** in deuterated chloroform ($CDCl_3$).

Position	Residue	$^{13}C^{a}$	$^{1}H^{a}$, multiplicity (J in Hz)	^{1}H-^{1}H COSY	^{1}H-^{13}C HMBC
2	C	138.12			
3	C	113.63			
4	CH	102.19	7.12, d (1.8)	6.67	131.30, 129.13, 113.63, 112.11
5	C	156.07			
6	CH	112.11	6.67, dd (8.8; 1.8)	7.12, 6.80	131.30, 102.19
7	CH	114.77	6.80, d (8.8)	6.67	156.07, 129.13
8	CH_3	13.17	2.49, s		138.12, 113.63
9	CH_2	74.87	5.11, s		172.81, 138.12, 129.13, 113.63
10	C	172.81			
1a	C	131.30			
3a	C	129.13			
5-OCH_3	CH_3	55.70	3.81, s		156.07
10-OCH_3	CH_3	56.90	3.44, s		172.81
1′	C	133.37			
2′/6′	CH	131.35	7.68, d (7.9)	7.48	168.31, 139.76, 131.35
3′/5′	CH	129.23	7.48, d (7.9)	7.68	139.76, 133.37, 129.23
4′	C	139.76			
7′	C	168.31			

[a] Chemical shifts in ppm.

Table S4. Data obtained from mono- and two-dimensional NMR analysis of **DBP3** in deuterated chloroform ($CDCl_3$).

Position	Residue	$^{13}C^{a}$	$^{1}H^{a}$, multiplicity (J in Hz)	^{1}H-^{1}H COSY	^{1}H-^{13}C HMBC
2	C	104.22			
3	C	71.92			
4	C	120.33			
5	C	152.74			
6	CH	114.36	6.84, d (9.1)	6.78	135.97, 120.33
7	CH	115.54	6.78, d (9.1)	6.84	152.74, 127.41
8	CH_3	21.69	1.96, s		104.22, 71.92
9	CH_2	40.43	4.14, d (18.2)	3.30	169.00, 127.41, 104.22, 71.92
			3.30, d (18.2)	4.14	169.00, 127.41, 104.22, 71.92
10	C	169.00			
1a	C	135.97			
3a	C	127.41			
5-OCH_3	CH_3	56.86	3.89, s		152.74
1′	C	133.65			
2′/6′	CH	130.19	7.67, d (81)	7.47	167.93, 138.79, 130.19
3′/5′	CH	129.31	7.47, d (8.1)	7.67	138.79, 133.65, 129.31
4′	C	138.79			
7′	C	167.93			

[a] Chemical shifts in ppm.

Table S5. Data obtained from mono- and two-dimensional NMR analysis of **DBP4** in deuterated chloroform ($CDCl_3$).

**Position**	**Residue**	**$^{13}C^{a}$**	**$^{1}H^{a}$, multiplicity (J in Hz)**	**^{1}H-^{1}H COSY**	**^{1}H-^{13}C HMBC**
2	C	148.70			
3	C	118.60			
4	CH	103.48	7.82, s		130.81, 127.20, 118.60, 114.16
5	C	157.35			
6	CH	114.16	6.74, s		157.35, 130.81, 127.20, 103.48
7	CH	114.50	6.74, s		
8	CH_3	12.80	2.78, s		148.70, 118.60
9	CH	185.99	10.34, s		148.70, 127.20, 118.60
1a	C	130.81			
3a	C	127.20			
5-OCH_3	CH_3	55.91	3.89, s		157.35
1′	C	132.25			
2′/6′	CH	131.85	7.72, d (8.8)	7.51	168.40, 141.15, 131.85
3′/5′	CH	129.69	7.51, d (8.8)	7.72	141.15, 132.25, 129.69
4′	C	141.15			
7′	C	168.40			

[a] Chemical shifts in ppm.

Table S6. Data obtained from mono- and two-dimensional NMR analysis of **DBP5** in deuterated chloroform ($CDCl_3$).

Position	Residue	$^{13}C^a$	$^1H^a$, multiplicity (J in Hz)	^1H-^1H COSY	^1H-^{13}C HMBC
2	C	133.52			
3	C	112.64			
4	CH	100.21	6.98, d (2.7)	6.73	129.64, 128.25, 113.27, 112.64
5	C	156.38			
6	CH	113.27	6.73, dd (9.1; 2.7)	6.98, 6.93	129.64, 100.21
7	CH	115.31	6.93, d (9.1)	6.73	156.38, 128.25
8	CH_3	13.15	2.40, s		133.52, 112.64
1a	C	129.64			
3a	C	128.25			
5-OCH_3	CH_3	55.74	3.87, s		156.38
1′	C	133.45			
2′/6′	CH	131.14	7.66, d (8.8)	7.49	167.87, 139.65, 131.14
3′/5′	CH	129.21	7.49, d (8.8)	7.66	139.65, 133.45, 129.21
4′	C	139.65			
7′	C	167.87			

[a] Chemical shifts in ppm.

Table S7. Data obtained from mono- and two-dimensional NMR analysis of **DBP6** in deuterated chloroform ($CDCl_3$).

Position	Residue	$^{13}C^{a}$	$^{1}H^{a}$, multiplicity (J in Hz)	^{1}H-^{1}H COSY	^{1}H-^{13}C HMBC
2	C	136.92			
3	C	116.01			
4	CH	101.59	7.07, d (2.5)	6.83, 6.69	130.93, 130.59, 116.01, 111.70
5	C	156.07			
6	CH	111.70	6.69, dd (9.0; 2.5)	7.07, 6.83	130.93, 101.59
7	CH	114.84	6.83, d (9.0)	6.69	156.07, 130.59
8	CH_3	13.16	2.44, s		136.92, 116.01
9	CH_2	64.84	4.60, s		136.92, 130.59, 116.01, 57.90
1a	C	130.93			
3a	C	130.59			
5-OCH_3	CH_3	55.71	3.85, s		156.07
9-OCH_3	CH_3	57.90	3.43, s		64.84
1′	C	133.81			
2′/6′	CH	131.21	7.68, d (8.4)	7.47	168.52, 139.37, 131.21
3′/5′	CH	129.12	7.47, d (8.4)	7.68	139,37, 133.81, 129.12
4′	C	139.37			
7′	C	168.52			

[a] Chemical shifts in ppm.

Table S8. Data obtained from mono- and two-dimensional NMR analysis of **DBP7** in deuterated chloroform ($CDCl_3$).

Position	Residue	$^{13}C^{a}$	$^{1}H^{a}$, multiplicity (J in Hz)	^{1}H-^{1}H COSY	^{1}H-^{13}C HMBC
1	C	134.94			
2	C	120.88			
3	CH	114.41	7.30, d (2.7)	7.26	195.89, 134.94, 121.27
4	C	154.62			
5	CH	121.27	7.26, dd (9.3; 2.7)	8.93, 7.30	134.94, 114.41
6	CH	122.80	8.93, d (9.3)	7.26	154.62, 120.88
7	CH_3	195.89			
8	CH_2	66.02	5.39, s		195.89, 170.38, 120.88
10	C	170.38			
11	C	20.58	2.28, s		170.38
4-OCH_3	CH_3	55.85	3.89, s		154.62
1′	C	133.14			
2′/6′	CH	128.90	7.98, d (8.5)	7.50	164.74, 138.27, 128.90
3′/5′	CH	129.11	7.50, d (8.5)	7.98	138.27, 133.14, 129.11
4′	C	138.27			
7′	C	164.74			

[a] Chemical shifts in ppm.

Table S9. Data obtained from mono- and two-dimensional NMR analysis of **DBP8** in deuterated chloroform ($CDCl_3$).

Position	Residue	$^{13}C^{a}$	$^{1}H^{a}$, multiplicity (J in Hz)	^{1}H-^{1}H COSY	^{1}H-^{13}C HMBC
2	C	135.74			
3	C	103.78			
4	CH	100.26	6.99, d (2.4)	6.81	127.95, 111.26, 103.78
5	C	155.85			
6	CH	111.26	6.81, dd (8.6; 2.4)	7.19, 6.99	127.95, 100.26
7	CH	111.04	7.19, d (8.6)	6.81	155.85, 130.13
8	CH_3	11.81	2.43, s		135.74, 103.78
9	CH_2	29.69	3.73, s		173.35, 135.74, 130.13, 103.78
10	C	173.35			
1a	C	127.95			
3a	C	130.13			
5-OCH_3	CH_3	55.93	3.87, s		155.85

[a] Chemical shifts in ppm.

Table S10. Data obtained from mono- and two-dimensional NMR analysis of **DBP9** in deuterated chloroform ($CDCl_3$).

Position	Residue	$^{13}C^{a}$	$^{1}H^{a}$, multiplicity (J in Hz)	^{1}H-^{1}H COSY	^{1}H-^{13}C HMBC
1	C	131.15			
2/6	CH	122.65	7.49, d (8.4)	6.93	157.26
3/5	CH	114.63	6.93, d (8.5)	7.49	131.15
4	C	157.26			
1′	C	133.79			
2′/6′	CH	129.45	7.85, d (8.2)	7.57	166.63, 138.45
3′/5′	CH	128.85	7.57, d (8.2)	7.85	133.79
4′	C	138.45			
7′	C	166.63			
4-OCH_3	CH_3	55.65	3.83, s		157.26

[a] Chemical shifts in ppm.
